# The Act of Measurement: The Influence of Behavioural Tests on Spider Fear and Disgust

**DOI:** 10.1007/s10862-025-10234-8

**Published:** 2025-07-21

**Authors:** Jacqueline Peters, Anne van Wonderen, Renée M. Visser, Merel Kindt

**Affiliations:** Department of Psychology, University of Amsterdam, Nieuwe Achtergracht 129 B, 1018 WS, Amsterdam, The Netherlands

**Keywords:** Psychological assessment, Spider fear, Disgust, Specific phobia, Avoidance behavior, Exposure

## Abstract

Behavioural assessments offer clinically relevant insights into anxious symptomatology, complementing self-report questionnaires in monitoring treatment effects. However, as a behavioural test is a form of brief exposure, it might not solely measure distress, but also influence it. In this study, we investigated whether measuring spider avoidance behaviour changes spider-related distress. Seventy-five individuals with a broad range of spider fear provided self-reported fear and disgust ratings, using the Spider Distress Scale, before and after engaging in a tarantula and in a house spider behavioural approach task (BAT). We found that self-reported fear, but not disgust, decreased after engaging in the behavioural assessments. A subsequent exploration within low- and high-fear subgroups showed that this pattern was driven by low-fear individuals, as in high-fear individuals spider fear and disgust remained unaffected by the behavioural assessments. Spider fear did not decrease on two exploratory questionnaires. In principle, these findings are promising for the validity of behavioural assessments in translational research where sub-clinical samples are typically targeted. However, they emphasise the need to carefully translate laboratory findings to (sub-)clinical populations, not only due to varying fear levels, but also because behavioural assessments may be experienced differently in the context of a treatment study.

## Introduction

Fear reactions in humans are traditionally categorised and assessed on three levels: behavioural, verbal (cognitive and affective), and physiological (Kozak & Miller, 1982; Lang, 1968; Mauss & Robinson, 2009). As avoidance behaviour maintains symptoms of anxiety disorders (Barlow, 2004; Craske et al., 2008; Krypotos et al., 2015), behavioural assessments, such as Behavioural Approach Tasks (BATs), are clinically relevant. They provide unique insights into approach-avoidance behaviour and are frequently used to monitor treatment effects (e.g., Lass-Hennemann & Michael, 2014; Michaliszyn et al., 2010; Muris & Merckelbach, 1996; Mystkowski et al., 2006; Olatunji et al., 2011; Peters et al., 2025; Soeter & Kindt, 2015). As such, behavioural assessments complement self-report measures, which are essential and practical in clinical science and practice to monitor symptom changes during treatment but that can be sensitive to social desirability biases (Furnham, 1986; Van de Mortel, 2008). However, as behavioural assessments necessarily involve a confrontation with the phobic cue, they may influence fear (behaviour), rather than just measure it (Lonsdorf et al., 2017; Rescorla, 1988). This unintended consequence may not be surprising given that behavioural assessments share key features with exposure treatments. Even though exposure treatments for long-lasting fears and phobias are generally longer, span multiple sessions, and discourage cessation, the core principle resembles that of behavioural assessments: both involve approaching phobic cues in a stepwise manner. Fear responses in sub-clinical samples, which are frequently utilised in translational research, may also change more readily when being confronted with the phobic cue during behavioural assessments as their underlying fear memories may be weaker than those in clinical populations (Elsey & Kindt, 2017). Exposure effects as a result of measuring avoidance behaviour would challenge the validity of behavioural assessments in translational research.

Spider fear serves as an ideal model to study the influence of behavioural assessments on self-reported fear expression, because it is common in the general population (Costello, 1982; Oosterink et al., 2009) and students (Seim & Spates, 2010), and it is limited to a narrow range of fear-evoking stimuli. This allows researchers to trigger and study the fear in experimental (e.g., Vansteenwegen et al., 2007) and clinical research settings (e.g., Peters et al., 2025), and to utilise standardised BATs to assess spider avoidance behaviour (e.g., Lass-Hennemann & Michael, 2014; Soeter & Kindt, 2015). Thus, even though the current study focuses on the effect of spider avoidance behaviour on spider-related distress, we anticipate that our findings hold transdiagnostic relevance for various fears and anxiety disorders.

Recent insights from our research group (Elsey & Kindt, 2021; Peters et al., 2025) and others (e.g., Siegel & Peterson, 2022) indicate that spider BATs may not only measure avoidance behaviour but might actively influence subjective experiences of fear. Also, the finding that merely observing pictures (Björkstrand et al., 2020) or videos (Vansteenwegen et al., 2007) of spiders was shown to reduce fear and behavioural avoidance in spider fearful individuals suggests that a confrontation with the phobic cue may be sufficient to affect fear. Previous findings thus raise critical questions about the potential influence of behavioural assessments on future avoidance behaviour and other aspects of fear expression.

Not only fear, but also disgust plays an important role in several anxiety disorders including spider phobia (Cisler et al., 2009; Davey, 2011; Olatunji et al., 2017). Disgust is clinically relevant as it uniquely motivates avoidance behaviour (Cisler et al., 2009; Davey, 2011; De Jong & Muris, 2002), and it also seems to be less susceptible to exposure effects than fear (Böhnlein et al., 2020; Olatunji et al., 2007b; Smits et al., 2002). Learned disgust was found to be – at least partially – resistant to unconditioned stimulus devaluation (Mertens et al., 2021) and extinction learning (i.e., the laboratory analogue to exposure) (Borg et al., 2016; Engelhard et al., 2014; Mason & Richardson, 2010), more so than learned fear (Mitchell et al., 2024; Olatunji et al., 2007a). Although the mechanisms underlying this limited responsiveness to change remain unclear, one possible explanation is that learned disgust operates as an evaluative process (Baeyens et al., 1988; Mertens et al., 2021), in which aversions – such as a dislike of spiders – are acquired through associations with disgust-eliciting and dirty environments. Since evaluative processes are more resistant to extinction than contingency learning (Baeyens et al., 1988; Díaz et al., 2005), the disgust-eliciting properties of the spider may still persist. Hence, even when spiders are no longer perceived as an immediate threat through extinction learning, they can still elicit avoidance behaviour if they continue to evoke feelings of disgust. Due to the clinical relevance of disgust in motivating avoidance and its resistance to exposure effects, we differentially assessed changes in spider fear and disgust before and after behavioural assessments by using the Spider Distress Scale (SDS; Peters et al., 2022), which we recently developed to reliably assess self-reported spider-related distress.

In the current study, we investigated whether measuring avoidance behaviour changes fear and disgust towards spiders in individuals with varying levels of spider fear. We expected that spider fear would decrease in response to behavioural assessments in this non-clinical sample, whereas we did not necessarily expect such a decrease in spider-related disgust. To test this hypothesis, we recruited 75 individuals with a broad range of spider-related distress who were not offered treatment and assessed their self-reported spider fear and disgust before and after engaging in two spider BATs, one involving a tarantula and one a house spider.

## Methods and Materials

### Participants

This study was completed by 75 participants (61 female; 14 male), aged 18 to 38 (*M* = 21.12, *SD* = 3.08), with varying levels of spider-related distress. Participants were recruited via the University of Amsterdam’s laboratory system, accessible to both students and the general population. Eligible participants were comfortable with English and had not previously taken part in research or therapy involving living spiders. This study was approved by the ethical review board of the University of Amsterdam (ID: 2020-COP-11923) and participants received 20 EUR or two psychology study credits for completing this study. Please note that we previously presented the baseline data of the current study in Peters et al. (study 4; 2022). The post-BAT data have not yet been reported.

### Materials and Measures

**Fig. 1:**
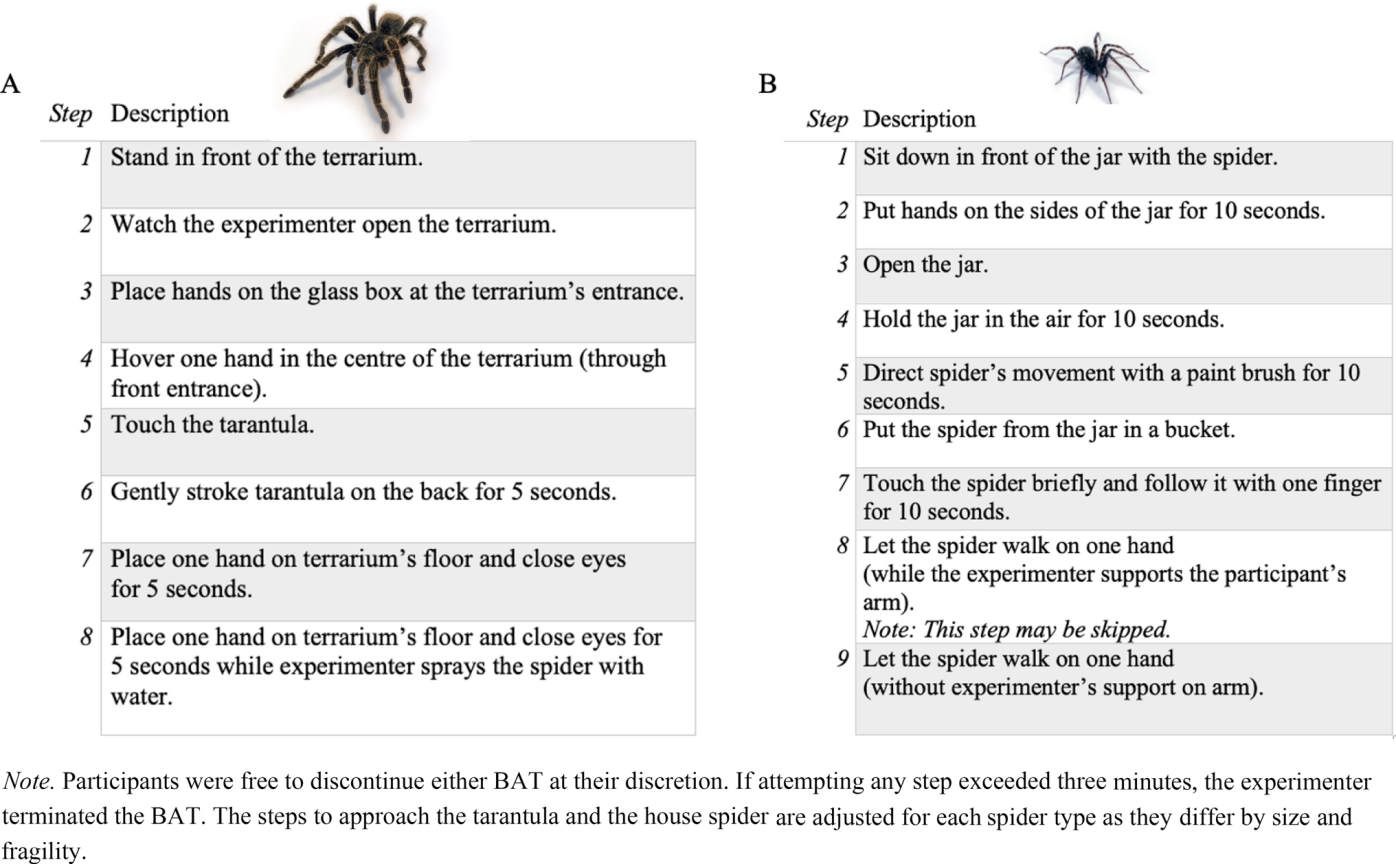
Tarantula (**A**) and House Spider (**B**) Behavioural Approach Task (BAT)

*Spider Behavioural Approach Tasks* (BATs; Peters et al., 2025). Participants were subjected to the 8-step tarantula BAT (TBAT; *grammostola porteri*, ~ 10 cm) and the 9-step house spider BAT (HBAT; *eratigena atrica*, ~ 3 cm) that has previously been used in treatment research as a generalization stimulus (e.g., Peters et al., 2025; Soeter & Kindt, 2015) in counterbalanced order (see Fig. 1). Participants were free to discontinue the BAT at any time and the experimenter terminated the task if the participant was unable to complete a step within three minutes. Participants also reported their distress levels and their peak fear/disgust (data not reported here, see https://osf.io/zdbyh/).

*Spider Distress Scale* (SDS; Peters et al., 2022). The SDS is a 17-item questionnaire to assess spider-related distress on two factors with a 7-point Likert scale. Thirteen items primarily target spider fear (SDS-F; range 0–78) and four items target spider-related disgust (SDS-D; range 0–24). The SDS and its subscales are reliable regarding internal consistency (McDonald’s ω ≥ 0.86) and the SDS has excellent test-retest reliability (*r* =.95) over the course of three weeks (study 3; Peters et al., 2022), as well as concurrent, convergent, and discriminant validity (study 4; Peters et al., 2022).

*Exploratory Questionnaires*. We used the Spider Phobia Questionnaire (SPQ; Klorman et al., 1974; range 0–31) and the Fear of Spiders Questionnaire (FSQ; Szymanski & O’Donohue, 1995; range 0-108) to explore changes in spider distress in addition to the SDS.

At baseline, we also administered the Disgust Propensity and Sensitivity Scale – Revised (DPSS-R; Fergus & Valentiner, 2009; van Overveld et al., 2010) and the State-Trait Anxiety Inventory (STAI-T; Spielberger et al., 1983), which were beyond the scope of the current study.

### Procedure

Participants were scheduled for their in-person session by phone, during which we conducted a structured clinical interview for DSM-5 specific phobias (SCID-5), modified to screen for spider phobia. For exploratory analyses, forty-one participants were categorised with sub-clinical spider fear and one participant fell into the spider phobia category (i.e., all DSM-5 criteria were fully met, including that the fear interferes with daily life or causes clinically significant distress) (American Psychiatric Association, 2013). These participants were pooled into the high-fear subgroup (*n* = 42) for exploratory analyses, whereas the remaining participants comprised the low-fear subgroup (*n* = 33).

During their in-person session, participants provided written informed consent, filled in several questionnaires including self-reported spider-related distress. Then, they engaged in the tarantula and in the house spider BAT in counterbalanced order^1^, and filled in the questionnaires on spider-related distress once again. All tasks were separated by 7-min breaks to minimise spill-over effects.

### Data Analysis

We used two-sided Bayesian dependent samples t-tests with the default Cauchy prior distribution with scale parameter set to $\:\frac{1}{\surd\:2}$ to assess the effects of the BATs on self-reported spider fear and disgust, using the SDS. We report Bayes Factors (BFs) for inference. BF_10_ > 1 indicates that the data are more likely under the model including a main effect than under the null model, whereas BF_10_ < 1 indicates that the data are most likely under the null model. We used non-parametric t-tests for stepwise behavioural data because these ranked data were not normally distributed and did not meet the assumptions for parametric tests. JASP (JASP Team, 2020) was used for Bayesian analyses and *ggplot2 *(Wickham, 2016) in R for data visualisation.

## Results

### Missing Data, Outliers, and Manipulation Checks

There were no missing data. We screened for extreme outliers exceeding three times the interquartile range (IQR) on the baseline SDS-F, SDS-D, SPQ, FSQ, and the total steps completed across BATs, but there were none. There were also no extreme outliers within the low- and high-fear subgroups. Additionally, we screened for normal outliers exceeding 1.5 times the IQR on our primary outcome variables SDS-F and SDS-D at baseline, and while there were none for the SDS-D, there was one outlier on the SDS-F within the high-fear subgroup (Participant ID: S06). We did not remove this outlier but checked for the robustness of our results by repeating relevant analyses without this participant.

We checked whether the order of the HBAT (*M*_*HBAT*_ = 8.03, *SD*_*HBAT*_ = 1.68; by subgroup: *M*_*HBAT Low Fear*_ = 8.67, *SD*_*HBAT Low Fear*_ = 1.11; *M*_*HBAT High Fear*_ = 7.52, *SD*_*HBAT High Fear*_ = 1.89) and TBAT (*M*_*TBAT*_ = 7.11, *SD*_*TBAT*_ = 1.64; by subgroup: *M*_*TBAT Low Fear*_ = 7.52, *SD*_*TBAT Low Fear*_ = 1.33; *M*_*TBAT High Fear*_ = 6.79, *SD*_*TBAT High Fear*_ = 1.80) affected changes in spider-related distress. The order of the BATs did not convincingly predict changes on the SDS-F (BF_10_ = 2.57; i.e., weak evidence for a main effect), and did not predict changes on the SDS-D (BF_10_ = 0.26) or the sum of steps completed in both BATs (Mann-Whitney: BF_10_ = 0.30) based on independent samples t-tests^2^.

For the exploratory analyses, we checked whether the high-fear subgroup (*n* = 42) showed more spider-related distress than the low-fear subgroup (*n* = 33). We found very strong evidence that high-fear individuals reported more spider fear (SDS-F; *M*_*Low Fear*_*=* 17.94, *SD*_*Low Fear*_*=* 11.75, *M*_*High Fear*_*=* 46.36, *SD*_*High Fear*_*=* 11.48; BF_10_ = 1.47 × 10^+13^) and disgust (SDS-D; *M*_*Low Fear*_*=* 9.82, *SD*_*Low Fear*_*=* 6.05, *M*_*High Fear*_*=* 15.36, *SD*_*High Fear*_*=* 5.49; BF_10_ = 244.11) than the low-fear subgroup. However, the evidence for less approach behaviour in the high-fear subgroup compared to the low-fear subgroup based on the summed number of steps completed in the TBAT and HBAT was anecdotal and thus not convincing (*M*_*Low Fear*_*=* 16.18, *SD*_*Low Fear*_*=* 2.33, *M*_*High Fear*_*=* 14.31, *SD*_*High Fear*_*=* 3.43; Mann-Whitney: BF_10_ = 1.58), possibly due to ceiling effects. In contrast, the evidence for this difference was moderate when using a parametric Student t-tests that did not account for the ranked nature of the stepwise BATs (BF_10_ = 5.00).

### Main Outcome Analyses: Changes in Spider Fear and Disgust

**Fig. 2:**
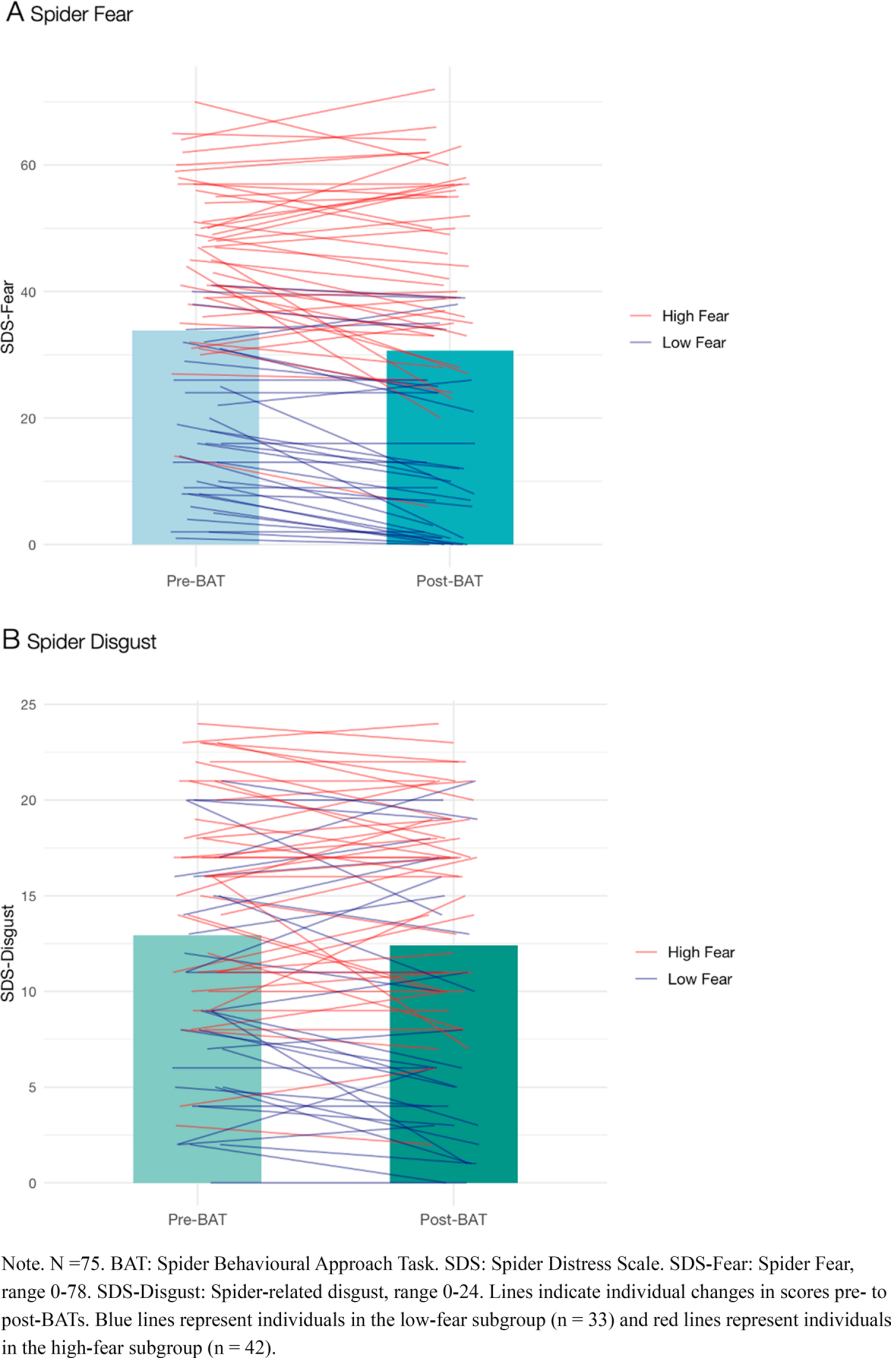
Spider fear (SDS-F) and disgust (SDS-D) before and after the Behavioural Approach Tasks

Using dependent samples Student t-tests, we found strong evidence that spider fear (SDS-F) decreased after undergoing the BATs (*M*_*Pre*_ = 33.85, *SD*_*Pre*_ = 18.29, *M*_*Post*_ = 30.69, *SD*_*Post*_ = 20.71; BF_10_ = 116.44; see Fig. 2a). Spider-related disgust (SDS-D) did not decrease (*M*_*Pre*_ = 12.92, *SD*_*Pre*_ = 6.34, *M*_*Post*_ = 12.41, *SD*_*Post*_ = 6.72; BF_10_ = 0.40; see Fig. 2b). These findings were robust as the pattern of the results remained similar when excluding the outlier, and when using non-parametric Wilcoxon signed-rank tests instead of Student t-tests.

#### Exploratory Analyses: Changes in Spider Fear and Disgust Within the Low- and High-Fear Subgroups

Within the low-fear subgroup, we found strong evidence that spider fear (SDS-F) decreased after undergoing the BATs (*M*_*Pre*_ = 17.94, *SD*_*Pre*_ = 11.75, *M*_*Post*_ = 13.67, *SD*_*Post*_ = 13.11; BF_10_ = 424.50). Spider-related disgust (SDS-D) did not decrease (*M*_*Pre*_ = 9.82, *SD*_*Pre*_ = 6.05, *M*_*Post*_ = 8.94, *SD*_*Post*_ = 6.71; BF_10_ = 0.69). Within the high-fear subgroup, spider fear (SDS-F; *M*_*Pre*_ = 46.36, *SD*_*Pre*_ = 11.48, *M*_*Post*_ = 44.07, *SD*_*Post*_ = 14.95; BF_10_ = 0.80) and disgust (SDS-D; *M*_*Pre*_ = 15.36, *SD*_*Pre*_ = 5.49, *M*_*Post*_ = 15.14, *SD*_*Post*_ = 5.38; BF_10_ = 0.19) did not change in response to the BATs. The pattern of these results remained similar upon removing the outlier, when using non-parametric tests, and when using a median split on the SDS-F instead of the SCID-5 to categorise individuals into the low-fear (SDS-F ≤ 36) and high-fear (SDS-F > 36) subgroups.

Note that a group by time interaction between the low- and high-fear subgroups regarding changes in spider fear would not have been interpretable due to the inherently non-random differences in spider-related distress at baseline. Thus, while we observed different patterns over time when independently assessing a fear reduction within subgroups, we cannot determine whether the reduction in spider fear after behavioural assessments in individuals with low fear was meaningfully stronger than the stable spider fear scores in the high-fear subgroup.

### Exploratory Analyses: Changes in the SPQ and FSQ

There were no detectable changes on the SPQ (*M*_*Pre*_ = 9.89, *SD*_*Pre*_ = 6.40, *M*_*Post*_ = 9.96, *SD*_*Post*_ = 6.37; BF_10_ = 0.13) and the FSQ (*M*_*Pre*_ = 35.44, *SD*_*Pre*_ = 24.54, *M*_*Post*_ = 34.39, *SD*_*Post*_ = 27.87; BF_10_ = 0.16) after the BATs, also when subgroups were analysed separately (BF_10_ ≤ 0.40). The results remained similar without the outlier and with non-parametric tests, except for a decrease on the FSQ in the low-fear subgroup when using non-parametric tests (BF_10_ = 21.54).

## Discussion

We examined changes in spider-related distress in individuals with a broad range of spider fear before and after they engaged in two behavioural assessments. Overall, individuals reported a reduction in spider fear, but not in spider-related disgust, which is in line with previous findings suggesting that disgust is more resistant to exposure effects than fear (Olatunji et al., 2007a; Smits et al., 2002). Whereas our findings show that behavioural assessments can reduce spider fear as observed in the full sample, this effect seemed to be driven by a fear reduction in people with low fear levels because no exposure effects were observed at the group level within our higher fear participants. Additionally, no exposure effects were found on the two exploratory questionnaires, the SPQ and FSQ. In principle, this finding is promising for the validity of behavioural assessments in translational research, as samples with elevated fear levels are typically utilised here to examine the effectiveness of treatment mechanisms. Our study affirms that pre-existing fear levels contribute to how individuals are affected by behavioural assessments. A potential mechanism for this phenomenon may be that individuals exhibiting lower fear levels tend to complete more steps during the behavioural assessments, and consequently may have greater opportunities for new learning, provided that they still experience some degree of distress towards spiders. People with higher fear levels may not only require completing more steps but would also need to be confronted with the phobic cue for an extended duration to initiate new learning, given their stronger underlying fear memories (Elsey & Kindt, 2017). However, behavioural assessments do not encourage individuals to remain in the feared situation when distress becomes intolerable, which means those with higher fear levels may not reach a point where they encounter anxiety-disconfirming information crucial for new learning (Craske et al., 2014; Rescorla & Wagner, 1972). This complexity emphasises the importance of carefully translating insights from experimental research with non-clinical or student samples to clinical research, instead of simply applying discoveries from the laboratory to the real world without rigorous replication and validation of measures in (sub-) clinical populations.

Although our findings indicate that exposure effects in response to behavioural assessments may not be a concern in samples with high fear levels, engaging in behavioural assessments in the context of a treatment study may be a different experience than measuring avoidance behaviour in isolation. For instance, individuals with phobias or other anxiety disorders tend to show high motivation to improve once they challenged their avoidance tendencies and enrolled in an intervention study or treatment. Sample characteristics in treatment-seeking samples, such as motivation and self-efficacy, may thus differ. In fact, merely believing to receive an active treatment can increase willingness and ability to approach the phobic cue, subsequently improving self-efficacy beliefs regarding future spider encounters (Siegel & Peterson, 2022). In our laboratory, it was noted that participants encountered difficulty distinguishing between fear evaluations and treatment within a preliminary intervention study, prompting them to exert greater effort during behavioural assessments (Elsey & Kindt, 2021). This heightened engagement may lead to increased exposure to the phobic stimulus, consequently offering learning opportunities instead of merely measuring fear behaviour. Difficulties differentiating treatment from assessment may occur within treatment studies as any confrontation with the phobic cue poses a challenge for phobic individuals. Lastly, once the fear is reduced after treatment, behavioural assessments may offer again an opportunity for new learning by allowing participants to test their fear behaviour and recognise their increased ability to approach the phobic cue, possibly further consolidating treatment effects (e.g., Peters et al., 2025). In terms of clinical implications, we thus recommend that the potential effects of behavioural assessments are considered when translating findings from treatment studies in clinical science to clinical practice, where any symptom improvement is desirable. It is a strength of the current study to first investigate the effects of measuring avoidance behaviour on spider-related distress independently, i.e., not within an intervention study where treatment-related factors may influence the results, but future research is encouraged to investigate the role of behavioural assessments within the treatment context. This next step could determine if behavioural assessments remain valid in treatment-seeking samples and to examine under which circumstances behavioural assessments contribute to treatment effects.

While the current study exhibits several strengths, including investigating the effects of behavioural assessments on self-reported fear independently and taking a dimensional approach by recruiting a sample with a broad range of spider fear and disgust, there are clearly also several limitations. First, the non-clinical nature of our sample resulted in relatively low spider distress levels among participants. This outcome may have been expected as individuals with high fear levels may be less inclined to participate in a study focussing on spider-related distress. Thus, our sample may be biased, and the inclusion of a clinical subgroup may have enhanced the generalizability of our results. However, including a clinical subgroup for exposure to their phobic cue in the present study, without offering any treatment, would have raised ethical concerns. Moreover, as we did not observe changes in spider-related distress among high-fear participants following the behavioural assessments, exposure effects may be even less likely in clinically phobic individuals. Therefore, the inclusion of a clinical subgroup was not prioritized in the current study, but should be considered in future research.

Second, the absence of a control group that did not engage in spider behavioural assessments prevents us from conclusively attributing the decrease in fear following the behavioural assessments to the confrontation with the phobic cue (e.g., Rescorla, 1988). It is possible that the observed decrease in self-reported fear could be due to *regression to the mean*, a statistical phenomenon where extreme values tend to approach the mean upon repeated measurement (Barnett et al., 2005; Bland & Altman, 1994). Regression to the mean seems unlikely in the current study however, as the variance in spider fear scores did not decrease from baseline until after behavioural assessments.

Third, unlike the SDS-F, our exploratory analyses with the SPQ and the FSQ did not indicate a reduction in spider fear, emphasizing that the observed reduction in self-reported fear was subtle. As the SDS differentiates unique elements of spider-related disgust from fear (Peters et al., 2022), it may be more sensitive to detect changes in fear than the SPQ and the FSQ. The SPQ is known to be less sensitive in the non-phobic range (Muris & Merckelbach, 1996), and it does not differentiate spider-related disgust from fear. While the FSQ is more (change-)sensitive in sub-clinical populations and was developed to complement the SPQ, it does not assess disgust, and we previously identified some ambiguous phrases in the FSQ that may challenge its reliability (see Peters et al., 2022).

## Conclusions

Measuring fear expression reliably over time is critical for a better understanding of fear learning and the evaluation of treatment developments. In the current study, we found a small decrease in spider fear, but not disgust, on the SDS after individuals with a broad range of spider-related distress engaged in two behavioural assessments. This effect seemed to be driven by individuals with low levels of fear, as we detected no exposure effects within our high-fear subgroup. Self-reported spider fear did not decrease on the exploratory SPQ and FSQ. These findings are promising for the validity of behavioural assessments in translational research, where individuals with sub-clinical fear levels are often targeted. However, they also highlight that such assessments may affect different populations in distinct ways, underscoring the need to carefully evaluate their validity when applying insights from experimental research to clinical contexts. This study represents an initial step towards understanding whether assessing avoidance behaviour influences the subjective experience of fear. Nevertheless, more controlled research with diverse samples is necessary to expand upon these findings. Finally, it remains unclear whether our findings extend to clinical treatment studies, where the treatment context itself may alter how individuals engage with and experience behavioural assessments.

## Data Availability

Anonymised data for confirmatory and exploratory analyses, R code for data visualisations, and supplementary materials can be found at the Open Science Framework web page for this study (https://osf.io/zdbyh/). Please note that we previously reported the baseline data presented in the current study in Peters et al. (study 4; 2022).

## Footnotes

1 Counterbalancing: Each participant performed two behavioural assessments (TBAT and HBAT) in a randomly assigned order. In intervention studies, this could reflect measuring approach-avoidance behaviour with the treatment stimulus (e.g., tarantula) and a generalization stimulus (e.g., house spider; e.g., Peters et al., 2025). We used random.org to randomize the order of behavioural assessments so that for every 10 participants, five started with the TBAT and five started with the HBAT.

2 We did not control for stepwise completion of the BATs when assessing changes in spider-related distress because the ability and willingness to show more approach behaviour is associated with individuals’ pre-existing spider fear levels (Cochrane et al., 2008; Peters et al., 2022), and is thus part of the effect that we were interested in.
